# Increased Walking Speed Reduces Hospitalization Rates in Patients with Cardiovascular Disease During Exercise-Based Secondary Prevention

**DOI:** 10.3390/jcm14134583

**Published:** 2025-06-27

**Authors:** Andrea Raisi, Tommaso Piva, Jonathan Myers, Valentina Zerbini, Erica Menegatti, Margherita Lembo, Sofia Michelon, Isabella Meneghini, Giovanni Grazzi, Gianni Mazzoni, Simona Mandini

**Affiliations:** 1 Center for Exercise Science and Sport, Department of Neuroscience and Rehabilitation, University of Ferrara, Via Gramicia 35, 44121 Ferrara, Italy; andrea.raisi@unife.it (A.R.); tommaso.piva@unife.it (T.P.); margherita01.lembo@edu.unife.it (M.L.); sofia.michelon@edu.unife.it (S.M.); isabella.meneghini@edu.unife.it (I.M.); giovanni.grazzi@unife.it (G.G.); gianni.mazzoni@unife.it (G.M.); simona.mandini@unife.it (S.M.); 2 Healthy Living for Pandemic Event Protection (HL-PIVOT) Network, University of Illinois at Chicago, Chicago, IL 60607, USA; drj993@aol.com; 3 Division of Cardiology, VA Palo Alto, Palo Alto, CA 94304, USA; 4 Department of Medicine, Stanford University School of Medicine, Stanford, CA 94305, USA; 5 Department of Environmental and Prevention Sciences, University of Ferrara, 44121 Ferrara, Italy; erica.menegatti@unife.it; 6 Public Health Department, AUSL Ferrara, 44121 Ferrara, Italy

**Keywords:** cardiovascular disease, hospitalization, walking speed, exercise, cardiac rehabilitation, secondary prevention

## Abstract

**Background/Objectives**: Walking speed (WS) is associated with morbidity and mortality. This study sought to investigate the associations between WS and hospitalization among patients with stable cardiovascular disease (CVD) and analyze how changes in WS impact all-cause hospitalization during exercise interventions. **Methods**: Of the 3328 patients in the ITER registry, 2871 (aged 65 ± 11 years) were analyzed. WS was measured using the 1 km treadmill walking test (1 km-TWT). Hospitalization was evaluated after one and three years according to the baseline WS tertiles. Additionally, 1465 patients were re-evaluated three years after the baseline, categorized into SlowWS and FastWS groups, and subsequently associated with changes in WS (worsening or low, moderate, and high improvements), generating six joint categories. Hospitalization was re-assessed during the fourth and sixth years after the baseline. The associations between WS and all-cause and CVD hospitalization were examined using Cox proportional hazard models, adjusting for demographic and clinical confounders. **Results**: A higher baseline WS was inversely associated with one-year hospitalization, with a 42% lower risk of all-cause hospitalization (95% CI: 0.51, 0.66) and a 38% lower risk of cardiovascular-related events (95% CI: 0.45, 0.86) compared to those in slower patients. Significant but mitigated magnitudes were observed for three-year hospitalization. A similar trend resulted in WS changes over time. Interestingly, the six-year risk in the SlowWS-high group was a 43% (95% CI: 0.45, 0.74) lower risk, which was comparable to that in the FastWS-low patients. **Conclusions**: The 1 km-TWT effectively predicts hospitalization among cardiac outpatients and is a valuable educational tool for exercise-based interventions in secondary prevention. These findings emphasize the efficacy of exercise-based programs, highlighting the importance of promoting exercise in long-term CVD management.

## 1. Introduction

Cardiovascular disease (CVD) remains the main cause of morbidity and mortality globally, accounting for about 17 million deaths annually [1]. As a consequence of advancements in the intervention strategies, more patients with CVD are surviving initial events. However, this better survival has led to a greater number of patients requiring long-term management, resulting in a growing social and economic burden for healthcare systems [2,3,4]. According to recent statistics, the annual cost of CVD in Europe, including treatment, medications, and hospital services, is estimated to be EUR 282 billion annually, corresponding to 11% of the total health expenditure within the European Union [5,6]. In this context, cardiac rehabilitation and secondary prevention programs have been highly recommended for managing the long-term continuum of care for patients and reducing healthcare costs [7,8,9].

Among the major risk factors and exercise parameters, walking speed (WS) has emerged as an important indicator of functional status [10]. It is strongly associated with exercise capacity and recognized as a potential additional “vital sign” [11]. A growing body of evidence suggests that WS reflects overall health; is positively related to independence and quality of life; and emerges as a predictor of adverse health outcomes [12,13,14]. In support of this, it has been demonstrated that WS is inversely associated with the hospitalization and mortality rates among older adults [15,16]. Several studies have sought to investigate the relationship between gait speed, hospitalization, and prognosis in patients with CVD, but these studies generally have been limited to small samples with specific diagnoses and relatively short walking tests [17,18,19]. Recent research was conducted by measuring moderate WS through an endurance treadmill walking test, highlighting its efficacy in measuring physical function and predicting the hospitalization and healthcare costs in patients with CVD [20,21,22]. Although these results supported the value of WS, they were conducted in small samples of male or female patients.

Thus, the aim of the current study was to provide further support for the application of WS by examining the relationship between WS, all-cause hospitalization, and disease-specific causes of hospitalization and how changes in WS impact all-cause hospitalization in a large cohort of outpatients with CVD involved in an exercise-based prevention program.

## 2. Materials and Methods

### 2.1. The Source and the Study Population

Data extracted from the ITER study (InTegrating exERcise into lifestyle of cardiac outpatients) were analyzed. ITER is an ongoing patient registry that evaluates the effectiveness of exercise-based secondary prevention programs in patients with stable CVD (ClinicalTrial.gov NCT05817305). The initial sample included 3328 participants enrolled between 1997 and 2023 at the Center for Exercise Science and Sport, the University of Ferrara, Italy. Patients were referred to the center by their general practitioner or a specialist doctor without limitations based on gender, ethnicity, or socioeconomic status. Activities were carried out by a multidisciplinary team comprising sport physicians, cardiologists, nurses, Sport Science professors, and junior researchers. Patients and caregivers received clear indications about the intervention, including understanding the need for a consensus and participation. All participants gave their written informed consent at the time of enrolment. This study was approved by the Ethics Committee of the University of Ferrara, cod. 105/2023/Oss/UniFe.

### 2.2. Measures

Cardiovascular disease history was defined as follows: acute myocardial infarction (AMI), percutaneous coronary intervention (PCI), coronary artery bypass graft, or heart valve repair or replacement. Coronary bypass graft surgery superseded other reasons for hospitalization. If the admitting diagnosis was AMI, it was coded as AMI regardless of whether a subsequent PCI was performed. If a PCI was performed in the absence of AMI, it was coded as PCI without AMI. If a valvular replacement was performed in the absence of AMI, it was coded as a valvular replacement. Admitting diagnoses of cardiac tumors, coronary artery anomalies, and heart transplantation were coded as other. Specific diagnoses were labeled according to the International Classification of Diseases coding system, version 10 (ICD-10). During each follow-up visit, the patients underwent a clinical evaluation including their medical history, a fasting blood analysis, and risk factor control. Their body mass index and blood pressure were measured, and hypertension was defined as a systolic blood pressure ≥140 mm Hg or a diastolic blood pressure ≥90 mm Hg [23]. During the center-based sessions, all patients completed a submaximal, moderate, and perceptually regulated 1 km treadmill walking test (1 km-TWT) [24,25]. Specifically, the patients started the test at a WS of 2.0 km/h, with a progressive increase of 0.3 km/h every 30 s up to a WS corresponding to a perceived exercise intensity of 11–13/20 on the Borg scale (6–20). The WS reached during this phase was relatively continuously maintained for 1000 m. During walking, WS was adjusted to a steady-state level of perceived effort allowing the patient to complete the test. The time taken to complete the 1000 m, along with heart rate and average and maximum WS, was recorded. Heart rate was monitored constantly using a Polar RS100 heart rate monitor (Polar Electro, Kempele, Finland). Patients unable to complete the test at a WS ≥ 3.0 km/h performed validated shorter forms over 500 m or 200 m [26,27,28]. In order to investigate the efficacy of the intervention over the follow-up time by analyzing the changes in WS, the difference between the first and last observations was calculated, and the results were categorized into three tertiles: low improvement or worsening (<0.4 km/h), moderate improvement (0.4–1.3 km/h), and high improvement (>1.3 km/h).

### 2.3. The Exercise Intervention

During each visit, an educational intervention aiming to reinforce the idea of maintaining a physically active lifestyle was conducted [29]. In addition, based on the results of the 1 km-TWT, a home-based exercise program was designed. The 1 km-TWT plays an important educational role, as patients are able to experience the proper intensity and duration of walking in such a way to reach a certain learning effect [29]. The training program was repeatedly individualized during each visit, aiming for 30 to 60 min of moderate-intensity aerobic activity such as brisk walking, for at least 5 days, and preferably 7 days, per week. Less motivated or less fit patients were encouraged to accumulate the daily recommended volume in shorter bouts of at least 10 min. Given the importance of generating a long-term improvement in exercise habits, patients were invited to engage in additional forms of leisure-time physical activity. However, due to the lack of consistent objectively measured physical activity levels, these were not included in the analyses.

### 2.4. Follow-Up and Hospitalization

All-cause and cardiovascular disease hospitalization was followed up in the patients. Information on hospitalization was provided by the regional Health Service Registry of the Emilia-Romagna region or acquired by contacting the patient’s general practitioner or relatives. The time from the baseline to the index hospitalization was calculated in months. The primary aim of this study was to investigate the all-cause and CVD hospitalization at one and three years. The secondary aim was to analyze the associations between hospitalization during the fourth and sixth years after the baseline and the WS changes, analyzed in a subgroup of patients re-evaluated three years after the baseline. For all-cause hospitalization, any hospital admission was considered an event, while those for cardiovascular disease were considered where CVD diagnoses were reported in the registry. Patients who had more than one hospital admission within a 24 h period, usually due to transferal to a second hospital, were classified as having had a single hospitalization. For patients experiencing more than one hospitalization, only the first event was considered in the analysis.

### 2.5. The Statistical Analysis

Descriptive statistics for continuous variables are presented as the mean and standard deviation or as the frequency and percentage for categorical variables. Their normal distribution was verified through a Kolmogorov–Smirnov test. The statistical comparison of the baseline characteristics was performed using a one-way ANOVA for continuous variables and the χ^2^ test for categorical variables. Differences in the annual hospitalization rates were investigated using the McNemar’s test. For the first analysis, the patients were categorized into three tertiles (slower, medium, and faster) based on their baseline WS, adjusting for age and sex to generate homogeneous groups. In order to investigate secondary endpoints, two new age–sex-specific quintiles of the baseline WS were created (SlowWS and FastWS) and subsequently associated with the change in WS tertiles, generating six joint categories (SlowWS-low, SlowWS-moderate, SlowWS-high, FastWS-low, FastWS-moderate, and FastWS-high). All associations were investigated by applying Cox proportional hazard models, and all results were reported as hazard ratios (HRs) and 95% confidence intervals (CIs). The Slower and SlowWS-low groups were considered reference categories. All associations were adjusted for confounders with three models that included an increasing number of covariates. Model 0 was unadjusted. Model 1 (the minimally adjusted model) was adjusted for age, sex, marital status, and education. Model 2 (the clinical variable model) was adjusted for all of the variables in model 1 plus body mass index, myocardial infarction, coronary artery bypass graft, family history, hypertension, diabetes, and dyslipidemia. Confounders were selected by investigating the relationships between outcome, exposure, and covariates through a Directed Acyclic Graph (DAG) model [30]. Based on the assumptions described in the diagram (Supplementary Figure S1), the minimal adjustment set was identified following the backdoor criterion [31]. A Schoenfeld residual analysis was performed to assess the assumption of proportionality. The proportional hazard assumption was met by all models. Nonlinear associations were explored using penalized cubic splines fitted into Cox proportional hazard models [32]. To reduce the potential influence of reverse causality, sensitivity analyses were performed by excluding all participants with more than three major cardiovascular risk factors. The threshold of >3 was selected based on clinical relevance and the previous literature identifying the presence of multiple comorbidities as a marker of frailty and a poor prognosis in patients with CVD. The level of statistical significance was set at *p* < 0.05. The statistical analyses were performed using R Statistical Software 4.5.1 [R Core Team. A language and environment for statistical computing. Published online 2021. https://www.R-project.org/].

## 3. Results

Of the 3328 patients, 2871 were included in this study. A total of 457 patients (13%) were excluded for the following reasons: (1) the inability to complete the test; (2) heart failure classified as class III or higher according to the New York Heart Association [33]; (3) other physical or psychological conditions that interfered with walking capacity; and (4) missing data for the measures or covariates considered in the analysis. A detailed flow chart of the participants included in the analyses is provided in Supplementary Figure S2. The 1 km-TWT was performed by all patients without any major complications. The average walking speed measured throughout the test was 4.1 ± 1.2 km/h. The demographic and clinical characteristics of the patients stratified by tertile of measured WS are presented in Table 1.

### 3.1. Association of Baseline WS with All-Cause and Cardiovascular Disease Hospitalization Risk

**Figure 1:**
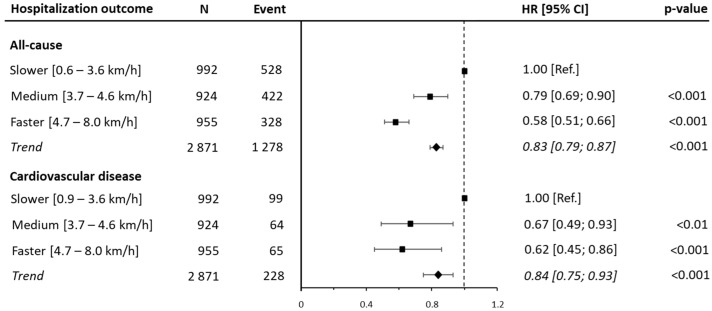
Associations of walking speed tertiles with 1-year all-cause and cardiovascular disease hospitalization. Data are presented as hazard ratios (HRs) and their 95% confidence intervals (CIs). The reference group was people reporting a lower range of walking speeds. Walking speed was analyzed both as a categorical variable and as a continuous variable. A hazard ratio for the trend was estimated and expressed as the risk per one unit increment in walking speed. Analyses were adjusted for age, sex, marital status, education, body mass index, myocardial infarction, coronary artery bypass graft, family history, hypertension, diabetes, and dyslipidemia.

During the first year following the baseline examination, 1278 patients (44.5% of the sample) were hospitalized, of whom 228 (7.9%) were hospitalized for CVD. After adjusting for confounders, a decreased risk of all-cause hospitalization was detected for medium WS (HR: 0.79 [95% CI: 0.69, 0.90; *p* < 0.001]) and faster WS (HR: 0.58 [95% CI: 0.51, 0.66]; *p* < 0.001]) patients as compared with that in the reference group. A similar magnitude was observed for hospitalization due to CVD (HR: 0.67 [95% CI: 0.49, 0.93; *p* < 0.01]; HR: 0.62 [95% CI: 0.45, 0.86; *p* < 0.001], respectively). In addition, each 1 km/h increment in the average WS was associated with a 27% reduction in all-cause hospitalization (HR: 0.83 [95% CI: 0.79, 0.87; *p* < 0.001]) and a 26% reduction in CVD hospitalization (HR: 0.84 [95% CI: 0.75, 0.93; *p* < 0.001]) (Figure 1 and Supplementary Table S1).

**Figure 2:**
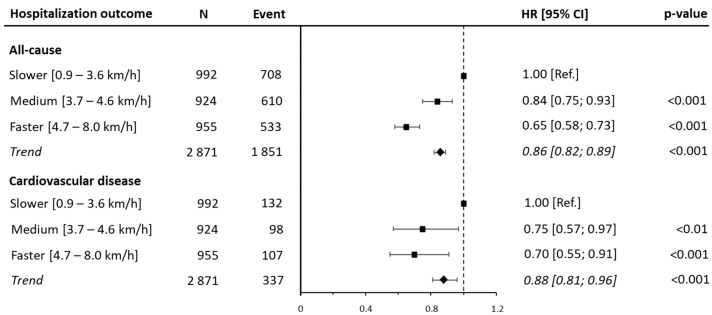
Associations of walking speed tertiles with 3-year all-cause and cardiovascular disease hospitalization. Data are presented as hazard ratio (HRs) and their 95% confidence intervals (CIs). The reference group was people reporting lower ranges of walking speeds. Walking speed was analyzed both as a categorical variable and as a continuous variable. A hazard ratio for trend was estimated and expressed as the risk per one unit increment in walking speed. Analyses were adjusted for age, sex, marital status, education, body mass index, myocardial infarction, coronary artery bypass graft, family history, hypertension, diabetes, and dyslipidemia.

This trend was confirmed after a secondary analysis considering the three-year hospitalization, where 1851 (64.4%) and 337 (11.7%) patients were hospitalized for all causes and CVD, respectively. The hospitalization risk was lower in the medium and faster categories for both all-cause (HR: 0.84 [95% CI: 0.75, 0.93; *p* < 0.001]; HR: 0.65 [95% CI: 0.58, 0.73; *p* < 0.001], respectively) and CVD hospitalization (HR: 0.75 [95% CI: 0.57, 0.97; *p* < 0.01]; HR: 0.70 [95% CI: 0.55, 0.91; *p* < 0.001], respectively), although the results were mitigated compared to those for 1-year hospitalization (Figure 2 and Supplementary Table S2).

**Figure 3:**
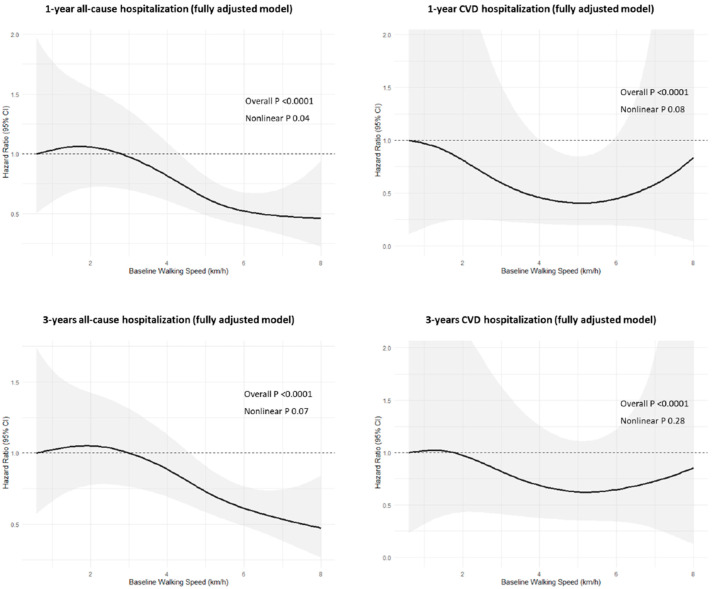
Nonlinear associations of walking speed with 1- and 3-year hospitalization due to all causes and cardiovascular disease. Models were adjusted for age, sex, marital status, education, body mass index, myocardial infarction, coronary artery bypass graft, family history, hypertension, diabetes, and dyslipidemia. Overall P indicates a linear trend, whereas nonlinear P indicates deviation from linearity.

When patients with the presence of more than three major risk factors were removed from the analysis, the associations remained unchanged (Supplementary Tables S3 and S4). Moreover, the relationship between WS and hospitalization is represented in Figure 3. It shows a nonlinear inverse association for 1-year hospitalization, while this appears to be linear in the other models. In all associations, there is a gradual decrease in risk starting from 3.0 km/h for all-cause hospitalization and from 1.5–2 km/h for hospitalization due to CVD.

### 3.2. Joint Associations of WS Changes with All-Cause Hospitalization Risk

**Figure 4:**
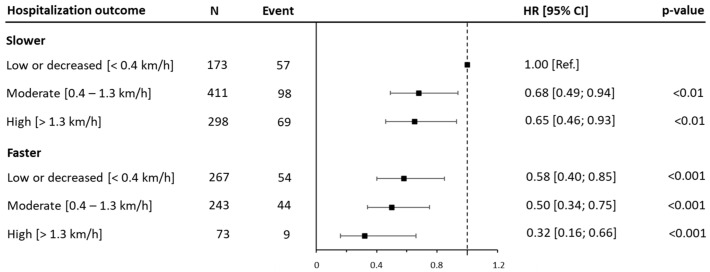
Joint associations of walking speed and variation in walking speed tertiles after three years with 4-year hospitalization. Data are presented as hazard ratios (HRs) and their 95% confidence intervals (CIs). The reference group was people reporting lower ranges of walking speeds. Change in walking speed is reported as tertiles (low improvement or worsening, moderate improvement, and high improvement) and abbreviated as low, moderate, and high. A hazard ratio for trend was estimated and expressed as the risk per one category increment in walking speed. Analyses were adjusted for age, sex, marital status, education, body mass index, myocardial infarction, coronary artery bypass graft, family history, hypertension, diabetes, and dyslipidemia.

Of the 2871 patients, 1465 (51.0%) were re-evaluated three years after the baseline and included in the analysis. Detailed information on the average WS at the baseline and after three years of follow-up, for each category, is shown in Supplementary Table S5. In terms of the hospitalization risk at four years (one year after the re-evaluation), a progressive reduction in risk was observed across the joint categories combining baseline walking speed and its improvement over time. Compared to the reference group (SlowWS-low), the patients in the FastWS-high group reported the lowest risk (HR: 0.32 [95% CI: 0.16, 0.66; *p* < 0.001]), while intermediate risk reductions were observed in the SlowWS-moderate, SlowWS-high, and FastWS-moderate groups (Figure 4 and Supplementary Table S6).

**Figure 5:**
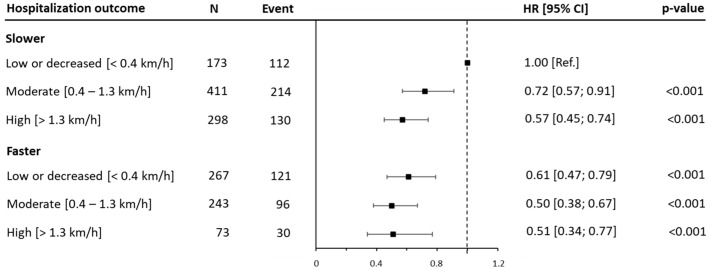
Joint associations of walking speed and variation of walking speed tertiles after three years with 6-years hospitalization. Data are presented as hazard ratios (HRs) and their 95% confidence interval (CIs). The reference group was people reporting lower ranges of walking speeds. Change in walking speed is reported as tertiles (low improvement or worsening, moderate improvement, and high improvement) and abbreviated as low, moderate, and high. A hazard ratio for trend was estimated and expressed as the risk per one category increment in walking speed. Analyses were adjusted for age, sex, marital status, education, body mass index, myocardial infarction, coronary artery bypass graft, family history, hypertension, diabetes, and dyslipidemia. WS, walking speed.

A similar trend was detected for the hospitalization risk at six years (three years after the re-evaluation), with the most notable difference between the FastWS-high patients (HR: 0.51 [95% CI: 0.34, 0.77; *p* < 0.001]) and the reference group (Figure 5 and Supplementary Table S7).

It is noteworthy that the SlowWS-high group demonstrated a slightly lower hospitalization risk (HR: 0.57 [95% CI: 0.45, 0.74; *p* < 0.001]) compared to that in faster individuals who reduced their walking speeds (HR: 0.61 [95% CI: 0.47, 0.79; *p* < 0.001]). Finally, the sensitivity analysis showed a similar magnitude, albeit improved, in the difference between the hospitalization risk among the joint categories for both the 4- and 6-year endpoints (Supplementary Tables S8 and S9).

## 4. Discussion

The current study included a cohort of 2871 patients with stable CVD over a wide range of ages and exercise capacities involved in an exercise-based secondary prevention program across a total of 25 years. The primary finding was an inverse association between the WS estimated by the 1 km-TWT and hospitalization.

These results are consistent with those of previous studies. For example, among 331 older patients with heart failure, an improvement in gait speed across tertiles was associated with a lower one-year rate of hospitalization due to heart failure (48.7%, 36.7%, and 25%, respectively) and all causes (71.3%, 58.6%, and 26.6%, respectively) [17]. Similarly, in 556 outpatients with stable coronary heart disease, each 104 m decrease in total walking distance, reflecting a lower gait speed, was associated with a 30% higher rate of cardiovascular events (HR: 1.30 [95% CI 1.10; 1.53]) [19]. These observations are similar to those in a Japanese study, in which 49 of 513 subjects experienced non-fatal cardiovascular events, and gait speed improvements were found to be a significant and independent predictor of cardiovascular events (HR: 0.71 [95% CI: 0.63; 0.81]) [18].

Another key finding was the inverse association between changes in walking speed over time and all-cause hospitalization. Patients who improved in their walking speed, particularly those starting at a higher baseline, had the lowest hospitalization risk during the follow-up. Interestingly, slow walkers who improved their walking speeds three years after the baseline (at a mean increase of 2.1 km/h) had a risk ratio that became comparable to that for faster walkers whose speeds did not improve or worsened (a mean decrease of 0.6 km/h). This analysis is partially consistent with previous studies. In a cohort of 1111 patients with CVD, those who achieved a moderate or high improvement exhibited lower rates of hospitalization (HR: 0.68 [95% CI 0.54; 0.86] and 0.58 [95% CI 0.45; 0.75], respectively) between the fourth and sixth years of follow-up, compared with those in the low-improvement reference group [20]. A similar trend was identified among 105 women with CVD re-evaluated three years after the baseline, in whom the hospitalization and mortality risk was progressively lower across tertiles for the moderate- (HR: 0.79 [95% CI: 0.46, 1.34]) and high-improvement (HR: 0.47 [95% CI: 0.25, 0.88] groups compared to that in the reference group [21]. Therefore, WS can be a useful, simple, and complementary tool for assessing the efficacy of secondary prevention programs. Previous studies have suggested several possible mechanisms that could explain the association of WS with all-cause hospitalization and particularly cardiovascular events. WS reflects the overall health of multiple organ systems, including cardiocirculatory, nervous, and musculoskeletal systems [18]. It is also influenced by frailty, comorbidities, and other age-related declines [34,35]. Hence, it is evident how these factors may contribute to modifying WS and an increasing hospitalization risk, especially in older patients with CVD, and the utility of WS assessment as a simple and comprehensive functional and prognostic tool is highlighted. In addition, the analyses showed a significant difference in the annual hospitalization rates. This trend appears to be consistent with previous research conducted in patients with CVD demonstrating a higher hospitalization rate following the first year of follow-up [36,37]. However, it is noteworthy that long-term participation in such interventions could contribute to reducing the overall risk of both all-cause and CVD hospitalization. Beyond physiological predictors, behavioral and psychosocial aspects likely played a role in shaping the walking speed trajectories and clinical outcomes. Although these factors were not directly measured, their potential influence—particularly in terms of motivation, perceived self-efficacy, and informal support systems—should be considered when interpreting the findings. The structured educational approach and personalized re-evaluations may have enhanced the adherence, but without objective tracking, the magnitude of this effect remains uncertain. Additionally, although this study was conducted in a real-world clinical setting, which supports the practical relevance of our findings, the single-center design and the relatively homogeneous patient population may limit the generalizability of its results. Future multicenter studies in more diverse populations are needed to confirm these associations.

### 4.1. Clinical Implications

These findings reinforce the usefulness of the 1 km-TWT in clinical practice. As mentioned above, walking tests of different distances and times have been used to assess exercise tolerance in various clinical conditions and among community-dwelling adults. Although these tests are submaximal, they often are too short or do not adequately reflect an individual’s functional capabilities since the participant is instructed to cover as much ground as possible or to walk as fast as possible for a certain time or distance [19,38]. Considering that daily physical activities rarely require maximal effort, assessments of submaximal exercise capacity through a moderate-endurance test could be useful for functionally evaluating patients and developing appropriate exercise prescriptions. In addition to its demonstrated prognostic contribution in prospective studies, this test is effective as an educational tool for exercise-based interventions within secondary prevention programs [29]. Moreover, the test is simple to perform, safer and suitable for patients with CVD, and adaptable to frail and older participants with limited mobility or mild cognitive deficits. Finally, it should be noted that, to the best of our knowledge, this is the first study that investigated the joint association between WS changes in patients with CVD and outcomes.

### 4.2. Limitations of This Study

The present study has several limitations. First, social, behavioral, and psychological factors independently associated with a reduced WS—such as depression, social support, or motivation—were not available for analysis. These unmeasured variables may have influenced both walking speed and hospitalization risk. Second, although adherence to physical activity was encouraged through individualized prescriptions and regular re-evaluations, the adherence rates were not directly monitored. This limits the ability to attribute changes in WS solely to the intervention. Third, given the significant loss of statistical power after categorization, the analysis of the WS changes after three years was only based on all-cause hospitalization. Fourth, there was a low percentage of women in the sample, which may influence the overall applicability of the results, albeit it is consistent with this specific population. Finally, the results were obtained from an ethnically homogeneous sample of patients that voluntarily participated in a secondary prevention program, indicating a need for external validation to generalize these findings.

## 5. Conclusions

The average WS estimated from a moderate and perceptually regulated treadmill walk test can predict all-cause and cardiovascular disease hospitalization. It also provides evidence that improvements in WS are proportionally associated with lower hospitalization rates. Overall, these findings underscore the health benefits of regular exercise and highlight the critical role of healthcare professionals in promoting exercise prescription as part of patient-centered secondary prevention programs.

## Figures and Tables

**Table 1:** The baseline characteristics of the 2871 patients examined by tertile of measured walking speed.

Variable	All Patients (n = 2 871)	Slower (n = 992)	Medium (n = 924)	Faster (n = 955)	*p*-Value
Measured walking speed (km/h)					
Mean (SD)	4.1 (1.2)	2.8 (0.6)	4.2 (0.3)	5.4 (0.6)	<0.0001
Range (min/max)	1.3–8.0	1.3–3.6	3.7–4.6	4.7–8.0	
Demographics					
Age (yr)	65 (11)	67 (10)	64 (10)	63 (11)	<0.001
Sex (women, n, %)	505 (18)	256 (26)	125 (14)	124 (13)	<0.001
BMI (Kg/m^2^)	27.4 (4.0)	27.6 (4.2)	27.5 (3.8)	27.2 (4.0)	0.078
LV ejection fraction (%)	58 (9)	57 (8)	58 (9)	58 (11)	<0.01
Marital status (married, %)	2296 (80)	767 (77)	765 (83)	764 (80)	<0.05
Education (high school, %)	1313 (46)	417 (42)	429 (46)	467 (49)	<0.01
Risk factors					
Family history (n, %)	1202 (42)	375 (38)	376 (41)	451 (47)	<0.001
Hypertension (n, %)	1745 (61)	660 (67)	518 (56)	567 (59)	<0.001
Diabetes (n, %)	501 (17)	201 (20)	134 (15)	166 (17)	<0.01
Current smoking (n, %)	639 (22)	200 (20)	200 (22)	239 (25)	<0.05
Hemoglobin (mg/dL)	13.5 (1.9)	13.4 (1.9)	13.5 (1.9)	13.6 (1.8)	0.060
Total cholesterol (mg/dL)	183 (48)	185 (51)	184 (45)	180 (46)	0.120
HDL cholesterol (mg/dL)	49 (15)	49 (15)	49 (16)	49 (14)	0.840
Serum triglycerides (mg/dL)	132 (69)	131 (70)	133 (66)	132 (70)	0.130
Serum creatinine (mg/dL)	1.07 (0.32)	1.09 (0.31)	1.08 (0.32)	1.05 (0.34)	<0.001
Medical history					
Myocardial infarction (n, %)	1399 (49)	484 (49)	473 (51)	442 (46)	0.100
PCI (n, %)	801 (28)	216 (22)	260 (28)	325 (34)	<0.001
CABG (n, %)	1110 (39)	427 (43)	365 (40)	318 (33)	<0.001
Valvular replacement (n, %)	481 (17)	205 (21)	143 (15)	133 (14)	<0.001
Other (n, %)	534 (19)	182 (18)	149 (16)	203 (21)	<0.05
Medications					
ACE inhibitor or ARB (n, %)	1673 (58)	572 (58)	535 (58)	566 (59)	0.740
Aspirin (n, %)	2105 (73)	656 (66)	678 (73)	771 (81)	<0.001
B-blocker (n, %)	1939 (68)	652 (66)	648 (70)	639 (67)	0.110
Calcium antagonist (n, %)	531 (18)	218 (22)	162 (18)	151 (16)	<0.01
Statin (n, %)	1788 (62)	537 (54)	582 (63)	669 (70)	<0.001
Diuretic (n, %)	670 (23)	362 (36)	188 (20)	120 (13)	<0.001

## Data Availability

The datasets used and/or analyzed during the current study can be made available by the corresponding author on reasonable request.

## Supplementary Materials

The following supporting information can be downloaded at https://www.mdpi.com/article/10.3390/jcm14134583/s1. Table S1: Age-sex-specific walking speed combinations and 1-year hospitalization; Table S2: Age-sex-specific walking speed combinations and 3-year hospitalization; Table S3: Association between age-sex-specific walking speed combinations and 1-year hospitalization conducted by excluding participants with the presence of more than 3 major risk factors; Table S4: Association between age-sex-specific walking speed combinations and 3-year hospitalization conducted by excluding participants with the presence of more than 3 major risk factors; Table S5: Mean difference between average walking speed at baseline and after three years of follow-up, for walking speed joint associations; Table S6: Association of age-sex-specific walking speed and variation of walking speed tertiles after three years with 4-years hospitalization; Table S7: Association of age-sex-specific walking speed and variation of walking speed tertiles after three years with 6-years hospitalization; Table S8: Association of age-sex-specific walking speed and variation of walking speed tertiles after three years with 4-years hospitalization conducted by excluding participants with the presence of more than 3 major risk factors; Table S9: Association of age-sex-specific walking speed and variation of walking speed tertiles after three years with 6-years hospitalization conducted by excluding participants with the presence of more than 3 major risk factors; Figure S1: Directed Acyclic Graph used to detect confounders of the relationship between walking speed and mortality; Figure S2: Flowchart of participants included in the study.

## Abbreviations

The following abbreviations are used in this manuscript:

CVD	Cardiovascular Disease
WS	Walking Speed
AMI	Acute Myocardial Infarction
PCI	Percutaneous Coronary Intervention
CABG	Coronary Artery Bypass Graft
DAG	Directed Acyclic Graph
1 km-TWT	1 km Treadmill Walking Test
BMI	Body Mass Index
HR	Hazard Ratio
CI	Confidence Interval
