# Supplementary material for: Increased Walking Speed Reduces Hospitalization Rates in Patients with Cardiovascular Disease During Exercise-Based Secondary Prevention
Source: J Clin Med. 2025 Jun 27;14(13):4583. doi: 10.3390/jcm14134583 (PMC12249556; doi:10.3390/jcm14134583)

Table S1: Age-sex-specific walking speed combinations and 1-year hospitalization

**All-cause hospitalization**												
	Model 0				*Model 1*				*Model 2*			
	HR	p-value	95% CI		HR	p-value	95% CI		HR	p-value	95% CI	
Slower	1.00	[ref]			1.00	[ref]			1.00	[ref]		
Medium	0.80	<0.001	0.71	0.92	0.78	<0.001	0.69	0.89	0.79	<0.001	0.69	0.90
Faster	0.56	<0.001	0.49	0.64	0.55	<0.001	0.48	0.64	0.58	<0.001	0.51	0.66
**Cardiovascular disease hospitalization**												
	Model 0				*Model 1*				*Model 2*			
	HR	p-value	95% CI		HR	p-value	95% CI		HR	p-value	95% CI	
Slower	1.00	[ref]			1.00	[ref]			1.00	[ref]		
Medium	0.66	<0.001	0.48	0.91	0.66	<0.001	0.48	0.90	0.67	<0.01	0.49	0.93
Faster	0.61	<0.001	0.45	0.84	0.61	<0.001	0.45	0.84	0.62	<0.001	0.45	0.86

Data presented as HR and their 95% CI. The reference group was people reporting lower range of walking speed. A hazard ratio for trend was estimated and expressed as risk per one category increment in walking speed. Analyses were adjusted for age, sex, marital status, education, body mass index, myocardial infarction, coronary artery bypass graft, family history, hypertension, diabetes, and dyslipidemia.

Table S2: Age-sex-specific walking speed combinations and 3-year hospitalization

**All-cause hospitalization**												
	Model 0				*Model 1*				*Model 2*			
	HR	p-value	95% CI		HR	p-value	95% CI		HR	p-value	95% CI	
Slower	1.00	[ref]			1.00	[ref]			1.00	[ref]		
Medium	0.84	<0.001	0.76	0.94	0.83	<0.001	0.74	0.92	0.84	<0.001	0.75	0.93
Faster	0.63	<0.001	0.56	0.70	0.62	<0.001	0.56	0.70	0.65	<0.001	0.58	0.73
**Cardiovascular disease hospitalization**												
	Model 0				*Model 1*				*Model 2*			

	HR	p-value	95% CI		HR	p-value	95% CI		HR	p-value	95% CI	
Slower	1.00		[ref]		1.00		[ref]		1.00		[ref]	
Medium	0.66	<0.01	0.48	0.91	0.73	<0.01	0.56	0.95	0.75	<0.01	0.57	0.97
Faster	0.61	<0.001	0.45	0.84	0.69	<0.001	0.53	0.90	0.70	<0.001	0.55	0.91

Data presented as HR and their 95% CI. The reference group was people reporting lower range of walking speed. A hazard ratio for trend was estimated and expressed as risk per one category increment in walking speed. Analyses were adjusted for age, sex, marital status, education, body mass index, myocardial infarction, coronary artery bypass graft, family history, hypertension, diabetes, and dyslipidemia.

Table S3: Association between age-sex-specific walking speed combinations and 1-year hospitalization conducted by excluding participants with the presence of more than 3 major risk factors.

**All-cause hospitalization**												
	Model 0				*Model 1*				*Model 2*			
	HR	p-value	95% CI		HR	p-value	95% CI		HR	p-value	95% CI	
Slower	1.00		[ref]		1.00		[ref]		1.00		[ref]	
Medium	0.77	<0.001	0.67	0.87	0.75	<0.001	0.66	0.85	0.77	<0.001	0.67	0.87
Faster	0.56	<0.001	0.48	0.64	0.54	<0.001	0.48	0.63	0.58	<0.001	0.50	0.67
**Cardiovascular disease hospitalization**												
	Model 0				*Model 1*				*Model 2*			
	HR	p-value	95% CI		HR	p-value	95% CI		HR	p-value	95% CI	
Slower	1.00		[ref]		1.00		[ref]		1.00		[ref]	
Medium	0.67	<0.01	0.48	0.93	0.66	<0.01	0.48	0.95	0.92	<0.01	0.48	0.94
Faster	0.61	<0.001	0.44	0.86	0.60	<0.001	0.44	0.90	0.95	<0.001	0.44	0.87

Data presented as HR and their 95% CI. The reference group was people reporting lower range of walking speed. A hazard ratio for trend was estimated and expressed as risk per one category increment in walking speed. Analyses were adjusted for age, sex, marital status, education, body mass index, myocardial infarction, coronary artery bypass graft, family history, hypertension, diabetes, and dyslipidemia.

Table S4: Association between age-sex-specific walking speed combinations and 3-year hospitalization conducted by excluding participants with the presence of more than 3 major risk factors.

**All-cause hospitalization**												
	Model 0				*Model 1*				*Model 2*			
	HR	p-value	95% CI		HR	p-value	95% CI		HR	p-value	95% CI	
Slower	1.00		[ref]		1.00		[ref]		1.00		[ref]	
Medium	0.81	<0.001	0.71	0.89	0.80	<0.001	0.71	0.89	0.81	<0.001	0.73	0.91
Faster	0.61	<0.001	0.54	0.69	0.61	<0.001	0.54	0.68	0.64	<0.001	0.57	0.72
**Cardiovascular disease hospitalization**												

	Model 0				*Model 1*				*Model 2*			
	HR	p-value	95% CI		HR	p-value	95% CI		HR	p-value	95% CI	
Slower	1.00		[ref]		1.00		[ref]		1.00		[ref]	
Medium	0.76	<0.01	0.57	0.99	0.75	<0.01	0.57	0.98	075	<0.01	0.57	0.99
Faster	0.71	<0.01	0.54	0.93	0.71	<0.01	0.54	0.93	0.71	<0.01	0.54	0.94

Data presented as HR and their 95% CI. The reference group was people reporting lower range of walking speed. A hazard ratio for trend was estimated and expressed as risk per one category increment in walking speed. Analyses were adjusted for age, sex, marital status, education, body mass index, myocardial infarction, coronary artery bypass graft, family history, hypertension, diabetes, and dyslipidemia.

Table S5: Mean difference between average walking speed at baseline and after three years of follow-up, for walking speed joint associations

**Categories**	**AvgWS_baseline**	**AvgWS_3years**
Slow WS-low	3.4 (0.7)<br>[1.3 - 4.4]	3.3 (0.8)<br>[1.2 - 4.3]
Slow WS-moderate	3.4 (0.7)<br>[1.2 – 4.4]	4.3 (0.8)<br>[1.9 – 5.6]
Slow WS-high	3.3 (0.8)<br>[0.9 - 4.4]	5.4 (0.8)<br>[3.2 - 7.5]
Fast WS-low	5.4 (0.6)<br>[4.5 - 8.0]	4.8 (0.9)<br>[0.6 - 7.4]
Fast WS-moderate	5.0 (0.4)<br>[4.5 - 6.7]	5.8 (0.5)<br>[4.9 – 7.2]
Fast WS-high	4.8 (0.3)<br>[4.5 – 5.6]	6.6 (0.6)<br>[5.8 -8.6]

Data are presented as mean (SD). Cut-offs are presented as [min-max]. Change in walking speed are reported as tertiles (low improvement or worsening, moderate improvement, high improvement) and abbreviated as low, moderate, and high. AvgWS, average walking speed

Table S6: Association of age-sex-specific walking speed and variation of walking speed tertiles after three years with 4-years hospitalization

**All-cause hospitalization**												
	Model 0				*Model 1*				*Model 2*			
	HR	p-value	95% CI		HR	p-value	95% CI		HR	p-value	95% CI	
Slow WS patients												
Low	1.00		[ref]		1.00		[ref]		1.00		[ref]	
Moderate	0.70	<0.01	0.51	0.97	0.69	<0.01	0.49	0.95	0.68	<0.01	0.49	0.94
High	0.67	<0.01	0.48	0.96	0.66	<0.01	0.46	0.93	0.65	<0.01	0.46	0.93

Fast WS patients												
Low	0.58	<0.001	0.40	0.84	0.57	<0.001	0.39	0.83	0.58	<0.001	0.40	0.85
Moderate	0.51	<0.001	0.34	0.75	0.49	<0.001	0.33	0.73	0.50	<0.001	0.34	0.75
High	0.34	<0.001	0.17	0.67	0.33	<0.001	0.16	0.65	0.32	<0.001	0.16	0.66

Data presented as HR and their 95% CI. The reference group was people reporting lower range of walking speed. Change in walking speed are reported as tertiles (low improvement or worsening, moderate improvement, high improvement) and abbreviated as low, moderate, and high. A hazard ratio for trend was estimated and expressed as risk per one category increment in walking speed. Analyses were adjusted for age, sex, marital status, education, body mass index, myocardial infarction, coronary artery bypass graft, family history, hypertension, diabetes, and dyslipidemia. WS, walking speed

Table S7: Association of age-sex-specific walking speed and variation of walking speed tertiles after three years with 6-years hospitalization

**All-cause hospitalization**												
	Model 0				*Model 1*				*Model 2*			
	HR	p-value	95% CI		HR	p-value	95% CI		HR	p-value	95% CI	
Slow WS patients												
Low	1.00		[ref]		1.00		[ref]		1.00		[ref]	
Moderate	0.70	<0.01	0.51	0.97	0.69	<0.01	0.49	0.95	0.72	<0.001	0.57	0.91
High	0.67	<0.01	0.47	0.96	0.65	<0.01	0.46	0.93	0.57	<0.001	0.45	0.74
Fast WS patients												
Low	0.58	<0.001	0.40	0.84	0.57	<0.001	0.39	0.83	0.61	<0.001	0.47	0.79
Moderate	0.51	<0.001	0.34	0.75	0.49	<0.001	0.33	0.73	0.50	<0.001	0.38	0.67
High	0.34	<0.001	0.17	0.67	0.33	<0.001	0.16	0.65	0.51	<0.001	0.34	0.77

Data presented as HR and their 95% CI. The reference group was people reporting lower range of walking speed. Change in walking speed are reported as tertiles (low improvement or worsening, moderate improvement, high improvement) and abbreviated as low, moderate, and high. A hazard ratio for trend was estimated and expressed as risk per one category increment in walking speed. Analyses were adjusted for age, sex, marital status, education, body mass index, myocardial infarction, coronary artery bypass graft, family history, hypertension, diabetes, and dyslipidemia. WS, walking speed

Table S8: Association of age-sex-specific walking speed and variation of walking speed tertiles after three years with 4-years hospitalization conducted by excluding participants with the presence of more than 3 major risk factors.

**All-cause hospitalization**												
	Model 0				*Model 1*				*Model 2*			
	HR	p-value	95% CI		HR	p-value	95% CI		HR	p-value	95% CI	
Slow WS patients												
Low	1.00		[ref]		1.00		[ref]		1.00		[ref]	
Moderate	0.65	<0.001	0.46	0.89	0.64	<0.001	0.46	0.89	0.64	<0.001	0.46	0.88

High	0.67	<0.01	0.47	0.94	0.65	<0.01	0.46	0.92	0.65	<0.01	0.45	0.93
Fast WS patients												
Low	0.58	<0.001	0.39	0.85	0.58	<0.001	0.39	0.84	0.59	<0.001	0.40	0.86
Moderate	0.49	<0.001	0.33	0.73	0.48	<0.001	0.32	0.71	0.49	<0.001	0.32	0.73
High	0.29	<0.001	0.14	0.61	0.29	<0.001	0.14	0.60	0.29	<0.001	0.14	0.61

Data presented as HR and their 95% CI. The reference group was people reporting lower range of walking speed. Change in walking speed are reported as tertiles (low improvement or worsening, moderate improvement, high improvement) and abbreviated as low, moderate, and high. A hazard ratio for trend was estimated and expressed as risk per one category increment in walking speed. Analyses were adjusted for age, sex, marital status, education, body mass index, myocardial infarction, coronary artery bypass graft, family history, hypertension, diabetes, and dyslipidemia. WS, walking speed

Table S9: Association of age-sex-specific walking speed and variation of walking speed tertiles after three years with 6-years hospitalization conducted by excluding participants with the presence of more than 3 major risk factors.

	**All-cause hospitalization**											
	Model 0				*Model 1*				*Model 2*			
	HR	p-value	95% CI		HR	p-value	95% CI		HR	p-value	95% CI	
Slow WS patients												
Low	1.00		[ref]		1.00		[ref]		1.00		[ref]	
Moderate	0.69	<0.01	0.55	0.87	0.69	<0.001	0.55	0.87	0.69	<0.001	0.55	0.88
High	0.56	<0.01	0.43	0.72	0.56	<0.001	0.43	0.72	0.56	<0.001	0.43	0.73
Fast WS patients												
Low	0.58	<0.001	0.44	0.75	0.58	<0.001	0.44	0.75	0.59	<0.001	0.45	0.77
Moderate	0.48	<0.001	0.36	0.62	0.47	<0.001	0.36	0.62	0.49	<0.001	0.37	0.65
High	0.47	<0.001	0.31	0.71	0.47	<0.001	0.31	0.70	0.48	<0.001	0.31	0.72

Data presented as HR and their 95% CI. The reference group was people reporting lower range of walking speed. Change in walking speed are reported as tertiles (low improvement or worsening, moderate improvement, high improvement) and abbreviated as low, moderate, and high. A hazard ratio for trend was estimated and expressed as risk per one category increment in walking speed. Analyses were adjusted for age, sex, marital status, education, body mass index, myocardial infarction, coronary artery bypass graft, family history, hypertension, diabetes, and dyslipidemia. WS, walking speed

Figure S1: Directed Acyclic Graph used to detect confounders of the relationship between walking speed and mortality

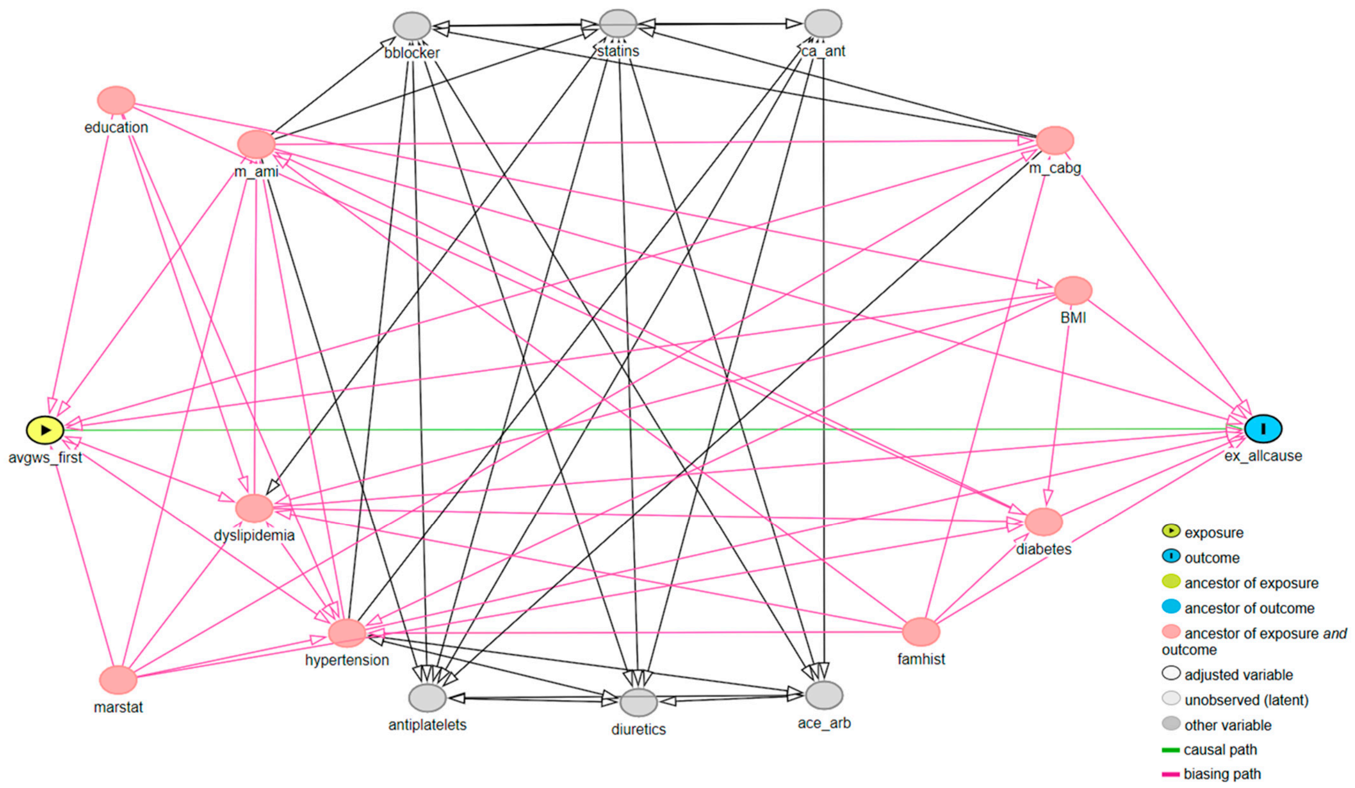

bblocker
statins
ca_ant
education
m_ami
m_cabg
BMI
avgws_first
ex_allcause
dyslipidemia
diabetes
hypertension
famhist
marstat
antiplatelets
diuretics
ace_arb
exposure
outcome
ancestor of exposure
ancestor of outcome
ancestor of exposure *and* outcome
adjusted variable
unobserved (latent)
other variable
causal path
biasing path

Figure S2. Flowchart of participants included in the study

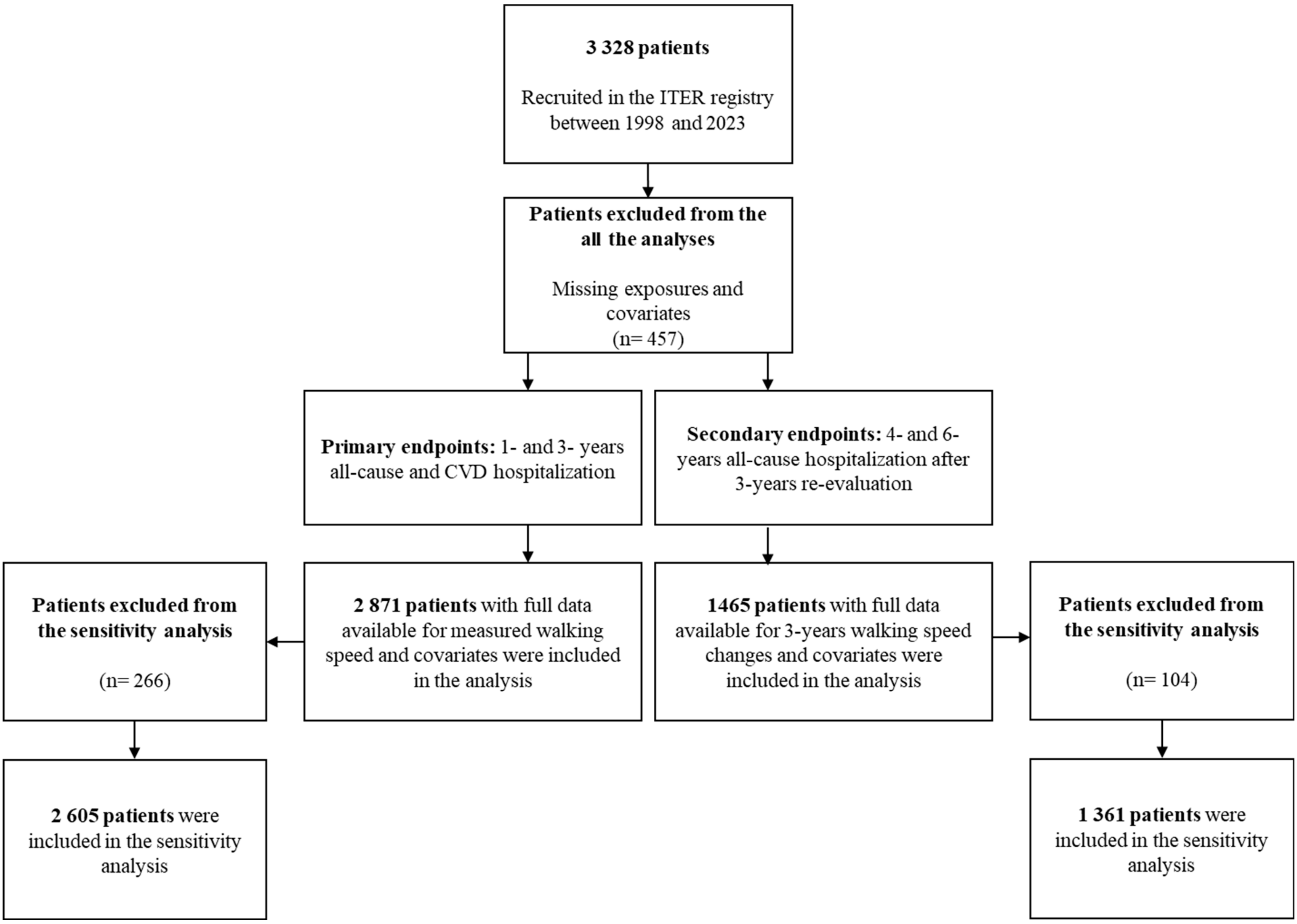

Supplement: Supplementary file 1 [file jcm-14-04583-s001.zip › jcm-3697714-supplementary.pdf]
